# Role of High-Resolution Chest Computed Tomography in a Child with Persistent Tachypnoea and Intercostal Retractions: A Case Report of Neuroendocrine Cell Hyperplasia

**DOI:** 10.3390/ijerph14101113

**Published:** 2017-09-25

**Authors:** Mara Lelii, Maria Francesca Patria, Raffaella Pinzani, Rossana Tenconi, Alessandro Mori, Nicola Bonelli, Nicola Principi, Susanna Esposito

**Affiliations:** 1 Pediatric Highly Intensive Care Unit, Università degli Studi di Milano, Fondazione IRCCS Ca’ Granda Ospedale Maggiore Policlinico, 20122 Milan, Italy; mara.lelii87@gmail.com (M.L.); f.patria@policlinico.mi.it (M.F.P.); raffaella.pinzani@hotmail.it (R.P.); rossana.tenconi@gmail.com (R.T.); alessandro.mori@studenti.unimi.it (A.M.); nicola.principi@unimi.it (N.P.); 2 Pediatric Radiology Unit, Fondazione IRCCS Ca’ Granda Ospedale Maggiore Policlinico, 20122 Milan, Italy; n.bonnic@gmail.com; 3 Pediatric Clinic, Department of Medical and Surgical Sciences, Università degli Studi di Perugia, Piazza Menghini 1, 06129 Perugia, Italy

**Keywords:** computed tomography, interstitial lung disease, neuroendocrine cell hyperplasia of infancy, respiratory distress, tachypnoea

## Abstract

*Background*: Chronic interstitial lung diseases in children (chILD) are a heterogeneous group of disorders that can represent a clinical challenge for pediatric pneumologists. Among them, neuroendocrine cell hyperplasia of infancy (NEHI) is a diffuse lung disease prevalent in the first years of life that spontaneously improves over time. The clinical presentation of NEHI is indistinguishable from other interstitial lung diseases, so a correct and non-invasive diagnosis by chest computed tomography (CT) without lung biopsy might not be simple. *Case presentation*: An 8-month-old male infant presented with a history of chronic tachypnoea and dyspnoea since 6 months of age. The patient was born at term, with APGAR scores of 9 and 10 at 1 and 5 min, respectively. Since his second month of life, the patient suffered from abnormal breathing, which was characterized by mild tachypnoea and costal retractions that worsened during breastfeeding, crying, and respiratory infections. Bilateral inspiratory crackles, preferential to the lung bases, without oxygen desaturation were detected. A chest X-ray showed a diffuse over-inflation of the lungs, but laboratory tests did not reveal any abnormalities. High-resolution chest CT documented patchy areas of ground-glass opacity involving the right upper lobe, middle lobe, and lingula, and showed mosaic areas of air-trapping, suggesting a diagnosis of NEHI. The infant was discharged without therapy and gradually improved over time. At 1 year of age, the patient was eupnoeic and chest auscultation had normalized. *Conclusions*: NEHI is an interstitial disease of infancy characterized by tachypnoea from the first months of life, with a good prognosis and for which a rational diagnostic approach is crucial for making a specific, early diagnosis. Initially, clinical suspicions can be confirmed with reasonable accuracy by a CT scan of the chest. Other more invasive and more expensive investigations should be reserved for selected cases that do not show a spontaneous, favourable clinical evolution.

## 1. Background

Chronic interstitial lung diseases (chILDs) in children are a heterogeneous group of disorders that can represent a clinical challenge for pediatric pneumologists due to the low frequency in the pediatric population, the complexity of the pathophysiological mechanisms, the different clinical profiles, the therapeutic management, and the extremely variable outcomes [1,2,3]. Although different classification systems have been proposed, the majority of chILDs are characterized by the presence of diffuse lung infiltrates on chest radiograph or computed tomography (CT) scan, usually with gas exchange impairment that leads to hypoxemia and hypercapnia. Tachypnoea, dyspnoea with costal retractions, cyanosis, chronic cough, exercise intolerance, failure to thrive, and inspiratory crackles are the typical clinical features; the severity of these features is related to the mechanisms underlying the development of the disease. In older children, a restrictive pulmonary function test is frequently observed. Several disorders are characterized by inflammation and fibrotic changes of the alveolar epithelium, although recently, under the wider definition of diffuse lung disease (DLD) (of which chILDs represent a subset), primary ciliary dyskinesia and infections have also been included [4,5,6].

According to the chILD Research Network (chILDRN) of the American Thoracic Society, these interstitial disorders are commonly divided into disorders more prevalent in infancy and disorders not specific to infancy. The first group includes diseases typical of infants and young children, while in the second category diseases more rarely represented in infancy and more common in adulthood are included [7]. Among the DLDs prevalent in infancy, neuroendocrine cell hyperplasia of infancy (NEHI) is a diffuse lung disease that spontaneously improves over time. The clinical presentation of NEHI is indistinguishable from the other interstitial lung diseases, so a correct and non-invasive diagnosis might not be simple. Nevertheless, the characterization of the disease has obvious implications in terms of patient management, therapeutic approach, and prognosis [8]. We report the case of an 8-month-old infant with persistent tachypnoea and retractions in whom a systematic diagnostic approach led to a correct NEHI diagnosis and appropriate management.

## 2. Case Presentation

An 8-month-old male infant presented to the Outpatient Clinic for Pediatric Pulmonary Diseases at the University of Milan, Fondazione IRCCS Ca’ Granda Ospedale Maggiore Policlinico, Milan, Italy, with a history of chronic tachypnoea and dyspnoea. He was the only child of non-consanguineous healthy parents and was born at term, with APGAR scores of 9 and 10 at 1 and 5 min, respectively, a birth weight of 3.820 kg (90th–97th percentile), and a length of 52 cm (90th percentile). The patient was exclusively breastfed up to 6 months of age with normal growth. Since his second month of life, the patient suffered from abnormal breathing, characterized by mild tachypnoea and costal retractions that worsened during breastfeeding, crying, and rhinitis.

**Figure 1:**
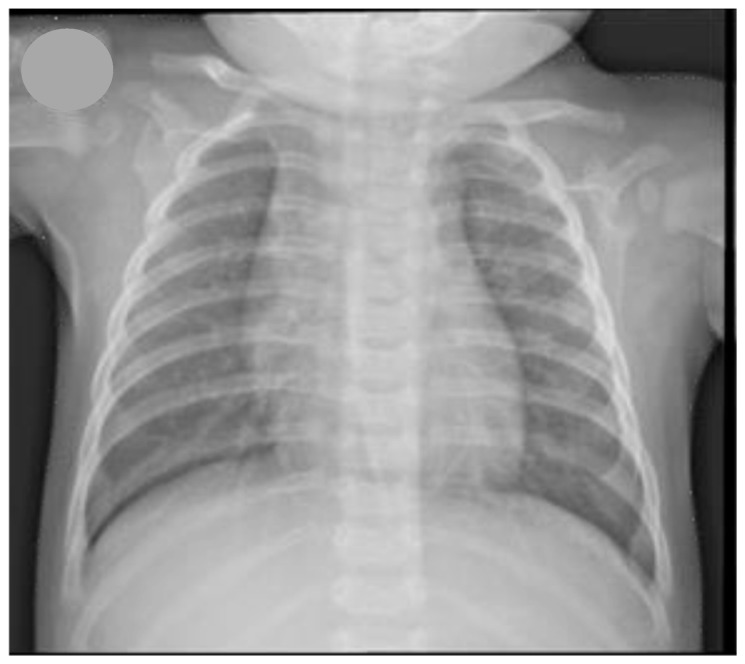
Chest X-ray showing a diffuse over-inflation of the lungs.

During his fourth month of life, he was admitted to the pediatric emergency department for an acute worsening of the dyspnoea. The child showed normal growth; his oxygen saturation on room air was 96%, no overnight or feeding desaturations were reported, his heart rate was 150 beats/min, and his respiratory rate was 70 breaths/min. A global reduction in breath sounds with bilateral inspiratory crackles and sporadic expiratory wheezing was heard. A chest X-ray showed a diffuse over-inflation of the lungs (Figure 1), but routine hematological and biochemistry blood tests did not reveal any abnormalities. The cardiologist documented a tiny patent ductus arteriosus, which was not hemodynamically significant. The infant was discharged with nebulized ipratropium bromide/salbutamol and an oral steroid (betamethasone), although these therapies many not have been necessary or beneficial. He was committed to the primary care pediatrician to monitor the clinical situation and decide whether further medical visits were needed. Drug treatment was continued without interruptions for two months. Later, considering the relatively good clinical state of the patient, all the therapies were suspended and they were administered occasionally only for few days when the child suffered from a likely viral respiratory infection with wheezing.

**Figure 2:**
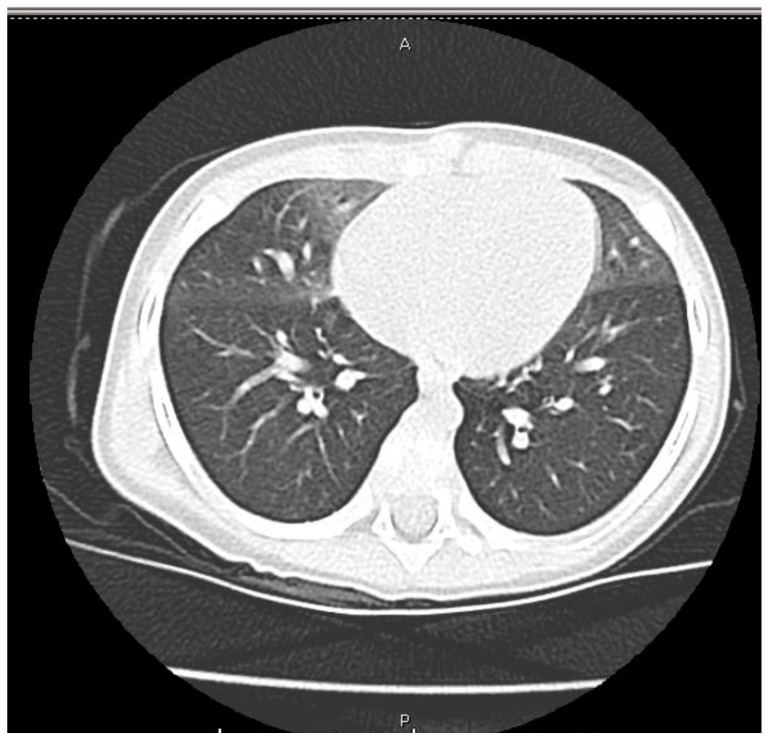
High-resolution chest computed tomography (CT) documenting patchy areas of ground-glass opacity involving the right upper lobe, middle lobe, and lingula, as well as mosaic areas of air-trapping, which are suggestive of a diagnosis of neuroendocrine cell hyperplasia of infancy (NEHI). A: anterior, P: posterior.

However, after four months, at 8 months of age, for the persistence of tachypnoea and sporadic wheezing in the absence of any desaturations, the patient was referred to our Outpatient Clinic and was subsequently hospitalized for an extensive diagnostic workup. Upon admission, clinical examination documented a body weight of 9 kg (50th–75th percentile), a height of 71 cm (50th–75th percentile), a respiratory rate of 60 breaths/min, an oxygen saturation on room air of 97%, and a heart rate of 135 beats/min. Bilateral inspiratory crackles, preferential to the lung bases, without oxygen desaturation were confirmed. A number of unnecessary investigations was performed. Blood tests revealed immunoglobulins and lymphocytes subpopulations, alfa 1-antitrypsin, C3 and C4 complement fractions, and thyroid function within normal limits. Evaluations for autoimmune diseases (i.e., anti-nuclear antibodies, anti-DNA antibodies, anti-neutrophil cytoplasmic antibodies, anti-smooth muscle antibodies, extractable nuclear antigen, anti-mitochondrial antibodies, anti-myeloperoxidase antibodies, and anti-proteinase 3 antibodies) were performed, all of which were within normal limits, except for a mild positivity of anti-cardiolipin IgG (40 GPL; range, 0–10 GPL). Serologic tests showed a positivity in IgM for *Coxsackievirus* and *Echovirus*, and a culture of nasopharyngeal secretions detected *Haemophilus influenzae* (10^6^ CFU/mL) and *Streptococcus pneumoniae* (10^5^ CFU/mL). Other serum biomarkers (i.e., Krebs von den Lungen-6, SP-A, SP-D, and lung tumor markers) were not tested. A sweat test and a cystic fibrosis genetic test were also normal, as was the genetic test for surfactant disorders (SP-C, SP-B, and ABCA3). Intraluminal impedance pH monitoring excluded acid or alkaline reflux, and a cardiac evaluation revealed the spontaneous resolution of the previously patent ductus arteriosus. Bronchoscopy showed a normal morphology of the airways and bronchoalveolar lavage showed a negative bacterial culture and negative viral polymerase chain reaction tests. A high-resolution chest CT documented patchy areas of ground-glass opacity involving the right upper lobe, middle lobe, and lingula, and showed mosaic areas of air-trapping (Figure 2), suggesting a diagnosis of NEHI.

Invasive diagnostic approach was avoided and the infant was discharged without therapy, gradually improving over time. At 1 year of age and at the latest follow-up (2 years of age), the patient was eupnoeic and chest auscultation had normalized.

### Ethics, Approval and Consent to Participate

Written informed consent for the publication of this case report and any accompanying images were obtained from the patient’s parents. A copy of the written consent is available for review by the Editor-in-Chief of this journal.

## 3. Discussion

NEHI is a childhood diffuse lung disease of unknown etiology, first recognized in 2005 [5,9,10]. NEHI is related to the distal airway hyperplasia of neuroendocrine epithelial cells, which produce vasoactive substances, especially bombesin. NEHI typically affects infants who are generally born at term and who present with nonspecific symptoms, such as longstanding tachypnoea, retractions, hypoxemia, and crackles on chest auscultation from the first months to the first few years of life [11]. The prevalence and incidence of NEHI remain unknown, although it seems to be a relatively rare disease. NEHI is a sporadic disease, but the rarely reported familial cases suggest that it might have a genetic component. However, to date, no mutation has been consistently identified [12,13]. The long-term outcome is generally favourable and for unknown reasons affected children gradually improve over time [14]. No clinical response to corticosteroids has been described.

Unfortunately, NEHI identification is not simple because the clinical features are not characteristic and often overlap those of other systemic lung diseases or chILDs [3,15,16]. This non-specific presentation obviously requires numerous evaluations to increase case recognition. In 2015, the European protocols for the diagnosis and initial treatment of interstitial lung diseases in children were published, with the aim of unifying standard operating procedures for chILDs [17]. According to this protocol, the preliminary diagnostic point is the identification of children who require further investigation by obtaining a careful medical history and recognizing clinical findings with their temporal occurrence. In particular, a systematic approach must include insights related to the family history, environmental exposure, neonatal period, age of onset, severity and length of symptoms, possible worsening during infections, and response to the therapy. Then, a careful examination should investigate the type and severity of respiratory symptoms. Clinical findings of NEHI include tachypnoea, hypoxemia, retractions, and crackles on auscultation, but there is intrasubject variability regarding the severity of the disease, which is related to the number of neuroendocrine cells in the distal airways. Indeed, in the most severe variants, failure to thrive and feeding problems are present. Wheezing is rarely described in the pediatric series, but can be present. In our patient, sporadic wheezing was repeatedly evidenced in the hospital and in the community. Moreover, Caimmi et al. described a 5-year-old child with a history of recurrent respiratory infections and wheezing, who presented with persistent hypoxemia and chronic respiratory symptoms, in whom after an extensive diagnostic work up for child, a diagnosis of NEHI was suggested [9]. However, the lack of univocal clinical signs of NEHI and the potential overlap with other diffuse lung diseases suggests the need for further diagnostic evaluations, such as blood tests for immune function, autoantibodies, complement fractions, and alpha 1-antitrypsin serum levels in any potential case. Chest X-ray is a common diagnostic tool, but the results are generally non-specific, and mostly show lung hyperinflation, which was the case with our patient. Intraluminal impedance pH monitoring is another generally advisable investigation, because gastro-oesophageal reflux is a common finding in DLD patients; however, it is unlikely that the reflux could play a primary role in the etiopathogenesis of the diseases. Cardiac evaluation and sweat tests are also suggested in the initial investigation. Lung function tests document a pattern of airway obstruction and air trapping, which is consistent with radiologic findings; however, the low specificity of the results and the complexity of the execution in infants make the test only slightly feasible in common clinical practice [5,17,18,19]. As in our case, high-resolution chest CT appears to be the most useful non-invasive imaging. According to Thacker et al. [10], although many CT findings are nonspecific and a definitive diagnosis usually cannot be reached by CT alone, the evidence of multi-lobar ground-glass opacity predominantly involving the right middle lobe and lingula as well as mosaic pattern of air-trapping are characteristics common in NEHI cases. In a series of 23 CT examinations of children with biopsy-proven NEHI and six CT scans of children with other chILDs, Brody et al. reported a CT sensitivity of at least 78% and a specificity of 100% in the diagnosis of NEHI [20]. Until a few years ago, because this condition is associated with increased numbers of neuroendocrine cells with no inflammatory characteristics and should be differentiated from other chILDs, lung biopsy and not bronchoalveolar lavage was considered the gold standard for the diagnosis [7,21]. Many authors consider the need for this invasive investigation debatable in children in good condition who have suggestive signs on the CT scan, as was the case with our patient. The patient had a mild expression of NEHI with no need for supplemental oxygen therapy, even during acute respiratory infections, and the CT scan was consistent with the diagnosis. Therefore, we decided to avoid other invasive procedures. In this context, genetic tests (i.e., cystic fibrosis and surfactant protein gene mutation investigations) and bronchoscopy with bronchoalveolar lavage should also be considered redundant. Generally, the need for further investigation, including lung biopsy, genetic tests, and bronchoscopy seems mandatory only in severely symptomatic children or if clinical and CT imaging findings are atypical [5,22,23].

## 4. Conclusions

Our case shows that NEHI is a part of a heterogeneous group of interstitial lung diseases of infancy characterized by chronic tachypnoea from the first months of life, with a good prognosis for which a rational diagnostic approach is crucial for making a specific early diagnosis and for avoiding invasive approaches such as lung biopsy. In mild to moderate cases, in the absence of progression in symptoms, clinical suspicion can be made with reasonable accuracy with a CT scan of the chest. Other more invasive and more expensive investigations should be reserved for selected cases associated with failure to thrive despite oxygen therapy, additional signs or symptoms in other organ systems, a family history of interstitial lung disease, or a deteriorating clinical course other than respiratory tract infections. In the future, follow-up data will permit the clarification of how to approach patients with NEHI according to their clinical findings.
